# Ameliorative Effect of Caffeic Acid on Capecitabine-Induced Hepatic and Renal Dysfunction: Involvement of the Antioxidant Defence System

**DOI:** 10.3390/medicines4040078

**Published:** 2017-10-25

**Authors:** Ebenezer Tunde Olayinka, Olaniyi Solomon Ola, Ayokanmi Ore, Oluwatobi Adewumi Adeyemo

**Affiliations:** Biochemistry Unit, Department of Chemical Sciences, Ajayi Crowther University, PMB 1066, Oyo, Oyo State 211213, Nigeria; ebenezertundeolayinka@gmail.com (E.T.O.); oreayokanmi@gmail.com (A.O.); dewumyt@gmail.com (O.A.A.)

**Keywords:** Caffeic acid, capecitabine, anti-oxidant, hepatoprotective, reactive oxygen species

## Abstract

**Background**: It has been postulated that during liver and kidney damage there is a decreased in the antioxidant status associated with a simultaneous increase in the reactive oxygen species and lipid peroxidation. In consonant with this, Capecitabine, an oral chemotherapy and inactive non-cytotoxic fluoropyrimidine considered for the treatment of advance colorectal cancer, has also been shown to induce oxidative stress in liver tissues. Caffeic acid, a typical hydroxycinnamic, has been claimed to be effective against oxidative stress. Therefore, this present work studied the protective effect of caffeic acid on oxidative stress-induced liver and kidney damage by the administration of capecitabine. **Methods**: Twenty-four male Wistar strain rats were randomly divided into four treatment groups: A. control, B. capecitabine (CPTB)-treated group (30 mg/kg b.w. CPTB), C. caffeic acid (CFA)-treated group (100 mg/kg b.w. CFA) and D. co-treated group with CFA (100 mg/kg b.w.) and CPTB (30 mg/kg b.w.). **Results**: Caffeic acid administration significantly ameliorated the elevated plasma biomarkers of hepatic and renal tissue damage induced by the capecitabine and improved enzymatic and non-enzymatic antioxidant levels in liver organ. **Conclusions**: The protective effect of caffeic acid could be attributed to its ability to boost the antioxidant defence system and reduce lipid peroxidation.

## 1. Introduction

Observable trends in the treatment of cancers of all kinds with synthetic chemotherapy are insufficient due to myriads of side effects that are associated with impaired organ function, most especially chemotherapy induced liver and kidney disorders. The liver is the most metabolically active tissue per unit weight and is therefore responsible for the majority of drug metabolism. Most metabolically active synthetic agents against cancer and tumour cells induce oxidative stress and caused injury to the tissues [1,2,3]. It is supposedly due to the additional off-target cytotoxic effect of most chemotherapeutic agents on healthy cells that effort have been made to synthesize prodrugs, like capecitabine, with chemotherapeutic effects.

Capecitabine, trade name Xeloda^®^, is a standard chemotherapy option considered for the treatment of advanced colorectal cancer (CRC) and also as an adjuvant therapy in colon cancer treatment [4,5]. It is an inactive non-cytotoxic fluoropyrimidine which becomes effective through its conversion to 5-fluorouracil (5-FU) in tumour cells [6]. During its biotransformation, it is converted through a series of enzymatic reaction steps to its effective cytotoxic form, 5-FU, preferentially in tumour tissue and also in the liver, by way of a three-step enzymatic cascade [7]. Therefore, due to this tumour-activated mechanism, more of the cancer-killing agent 5-FU is produced in cancer cells where it is needed, rather than in healthy cells. Despite the tumour-activated mechanism of its conversion to its effective form, administration of capecitabine had been reported to cause hand-foot syndrome and diarrhoea in humans [8,9], liver injury in rats [10], and hypertriglyceridaemia and coronary artery vasospasm in routine clinical practice [11,12]. Capecitabine has also been shown to lead to liver damage in rats through oxidative stress [10,13].

**Figure 1:**
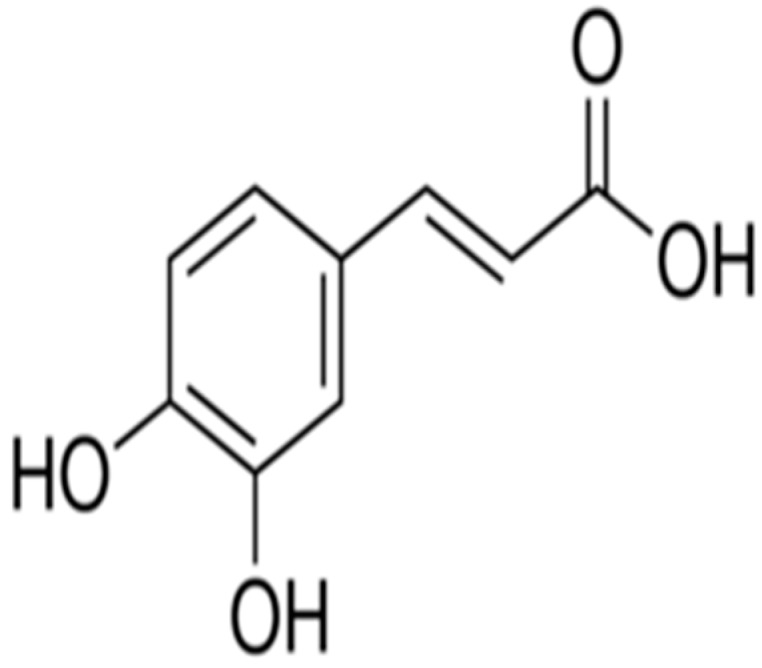
Caffeic acid.

In the current screening for novel therapeutic compounds, phenolic acids, which are widely distributed in plants [14], are very important because of their healthy interactions with numerous biological molecules. Caffeic acid with the structural formula displayed in Figure 1 is a phenolic acid and a typical hydroxycinnamic that has been claimed to be effective against oxidative stress and in blocking the generation of reactive oxygen species and xanthine/xanthine oxidase systems [15,16]. Several studies revealed that caffeic acid showed numerous properties that are related to its pharmacological and biological importance, such as immunomodulatory activities [17], anticarcinogenic effects [18], anti-inflammatory effects [19] and neuronal cell protection against oxidative stress [20]. It inhibited tumour promoter-mediated oxidative processes in the Hela cells’ culture [21]. It uses diverse mechanisms, such as ABTS(+) scavenging, DPPH scavenging, superoxide anion radical scavenging and ferrous ion chelating activities, in its antioxidant activities [22,23].

Thus, the aim of this study was to determine the possible protective effect of caffeic acid on capecitabine-induced tissue injury in rats using the antioxidant indices and markers of liver and kidney damages. This study may enhance the capecitabine regimen so that it could be well tolerated with few or minimal adverse effects.

## 2. Materials and Methods

### 2.1. Chemicals and Reagents

The substances used include the following: Capecitabine tablets (Xeloda^®^), a product of Rochie Product Ltd., Castle Hill, Australia; Glutathione (GSH), 1-chloro-2, 4-dinitrobenzene (CDNB), 5’,5’-dithio-bis-2-nitrobenzoic acid (DTNB), thiobarbituric acid (TBA), epinephrine and hydrogen peroxide were purchased from Sigma^®^ Chemical Company (London, UK); alanine aminotransferase (ALT), aspartate aminotransferase (AST), alkaline phosphatase (ALP), gamma-glutamyl transpeptidase (GGT), Urea, creatinine and bilirubin kits were obtained from RANDOX^®^ Laboratories Ltd., Antrim, UK. All other chemicals and reagent were of analytical grade and were obtained from British Drug House, Poole, UK.

### 2.2. Experimental Animals

Twenty four adult male Wistar rats weighing between 180–200 g were used for this research. The animals were purchased from the Department of Veterinary Medicine at the University of Ibadan and acclimatized for three weeks in the animal house of the Department of Chemical Sciences, Ajayi Crowther University, Oyo. They were kept in wire mesh cages, fed with commercial rat chow (Ladokun feeds Nigeria Ltd., Ibadan, OY, Nigeria) and supplied with water *ad libitum*. No anaesthesia was involved in this experiment and the protocol conformed to the guidelines of the National Institute of Health for laboratory animal care and use [24] with ethical approval code of Fns/Erd/201700005 from Faculty of Natural Sciences Ethical review committee of Ajayi Crowther University on 5 January 2017.

### 2.3. Animal Grouping and Drug Treatments

A simple randomized design was employed in this research study. After the adaptation period, the animals were randomly assigned to four main experimental groups of six animals each. The drug doses and antioxidant were administered once daily by oral gavage using oral intubator, as shown in the Table 1.

### 2.4. Collection of Blood and Liver Samples

The blood samples were collected from each animal 24 h after the final treatments, through the retro orbitals plexus into the lithium heparinized tubes. Animals were thereafter sacrificed and the liver was carefully excised from each animal in preparation for the cytosolic fraction.

### 2.5. Preparation of Plasma and Cytosolic Fractions

Each blood sample was centrifuged at 4000 rpm for 5 min using a CENCOM^®^ bench centrifuge (Analytika, Athens, Greece) in order to obtain the plasma. The plasma was stored at −4 °C for subsequent plasma assays. The excised liver from each rat was blotted free of blood stains, rinsed in ice-cold 1.15% KCl and homogenized in 4 volumes of ice-cold 0.01 M potassium phosphate buffer with pH 7.4. The liver homogenates were centrifuged at 12,000× *g* for 15 min at 4 °C using a refrigerated centrifuge from Eppendorf, UK Ltd., Stevenage, UK, and the post-mitochondrial fractions (PMF) were aliquoted and used for subsequent biochemical assays.

### 2.6. Determination of Plasma and Liver Protein Content

The biuret method of Gornall et al. [25] was used to determine protein concentration in the plasma and liver PMF. The reaction mixture consisted of 4 mL of biuret reagent and 1 mL of appropriately diluted sample. A blank mixture was prepared with 4 mL of biuret solution and 1 mL of distilled water. The protein concentration in the samples was extrapolated from the standard bovine serum albumin (BSA) curve.

### 2.7. Assay of Biomarkers of Hepatic and Renal Toxicity

Biomarkers of hepatotoxicity (total bilirubin (TBILI) level, and activities of alanine aminotransferase (ALT), aspartate aminotransferase (AST), and alkaline phosphatase (ALP)) and of renal toxicity (urea and creatinine) in the plasma were assayed using RANDOX^®^ diagnostic kits based on the manufacturer’s procedure. Assay of the TBILI level was based on Tietz et al.’s dimethyl sulphoxide method, 1994 [26]. The dimethyl sulphoxide formed a coloured compound with a maximum absorption at 550 nm. ALP activity was determined in accordance with Tietz’s principles [26]. The *p*-nitrophenol formed by the hydrolysis of *p*-nitrophenyl phosphate conferred a yellowish colour on the reaction mixture and its intensity was monitored at 405 nm to give a measure of enzyme activity. Determination of plasma ALT and AST activities was based on the principle described by Reltman and Frankel in 1957 [27]. ALT activity was measured by monitoring the concentration of pyruvate hydrazone formed with 2,4-dinitrophenylhydrazine at 546 nm. AST activity was measured by monitoring the concentration of oxaloacetate hydrazone formed with 2,4-dinitrophenylhydrazine at 546 nm, and gamma-glutamyl transpeptidase activity was determined following the principle described by Szasz [28].

### 2.8. Assay for Non-Enzymatic Antioxidants in the Liver

#### 2.8.1. Hepatic Reduced Glutathione Level

Jollow et al.’s method [29] was used to evaluate the level of hepatic GSH in the liver. The chromophoric product resulting from the reaction between Ellman’s reagent, 5,5′-dithiobis-(2-nitrobenzoic acid) and reduced glutathione possesses a molar absorption at 412 nm.

#### 2.8.2. Hepatic Ascorbic Acid (AA) Level

Jagota and Dani’s method [30] was used to determine the concentration of AA in the liver PMF. In this method, the interaction of AA in biological samples with Folin–Ciocalteu reagent gives a blue colour with maximum absorbance at 760 nm.

### 2.9. Assay of Hepatic Antioxidant Enzymes

#### 2.9.1. Hepatic Glutathione *S*-Transferase (GST) Activity

The activity of hepatic GST was determined by adopting the method described by Habig et al. [31]. Briefly, the assay mixture (3 mL) was made up of 150 μL of CDNB (3.37 mg/mL), 30 μL of reduced GSH (0.1 M), 2.79 mL phosphate buffer (0.1 M, pH 6.5) and 30 μL of liver PMF. The absorbance was measured at 340 nm, against the blank, after the reaction had stabilized for 60 s.

#### 2.9.2. Hepatic Superoxide Dismutase (SOD) Activity

The superoxide dismutase activity in the liver was determined by Misra and Fridovich’s procedure [32] in which the inhibition of the auto-oxidation of epinephrine at an alkaline medium pH 10.2 was monitored. One unit of SOD activity is defined as the amount of SOD necessary to cause 50% inhibition of the oxidation of adrenaline to adrenochrome over an interval of one minute.

#### 2.9.3. Hepatic Catalase Activity

The method described by Singha [33] was used to determine the hepatic catalase activity based on the reduction of dichromate in acetic acid to chromic acetate when heated in the presence of hydrogen peroxide (H_2_O_2_). The chromic acetate produced was measured spectrophotometrically at 570 nm and the amount of H_2_O_2_ remaining was extrapolated from the standard curve for H_2_O_2_. Catalase activity in the sample was expressed as micromoles of H_2_O_2_ consumed per min per mg protein.

### 2.10. Assay of Hepatic Level of Lipid Peroxidation

The degree of lipid peroxidation (LPO) in the liver was determined as described in Vashney and Kale’s method [34]. The method involved the reaction between malondialdehyde (MDA) and thiobarbituric acid to give a stable pink chromophore with maximum absorption at 532 nm. Lipid peroxidation, in nmole/mg protein, was computed as:

MDA (units/mg protein) = (Absorbance × volume of mixture)/(E_532nm_ × volume of sample × mg protein)

where E532 is the molar extinction coefficient for MDA = 1.56 × 105 M^−1^ cm^−1^.

### 2.11. Statistical Analysis

Data are presented as the means ± standard deviation (SD) of six replicates. Statistical significance was determined by a one-way analysis of variance (ANOVA), followed by Duncan’s multiple comparison between control and treated rats in all groups using the SigmaPlot^®^ statistical package (Systat Software Inc., San Jose, CA, USA). *p*-values of less than 0.05 (*p* < 0.05) were considered statistically significant.

## 3. Results

### 3.1. Influence of Caffeic Acid on Capecitabine-Induced Changes in Hepatic and Renal Function Markers in the Plasma of Rats

Capecitabine significantly increased the level of AST, ALT and ALP (Table 2) by 37.86%, 54.13% and 33.40% respectively, when compared with the control group (*p* < 0.05). Similarly, administration of capecitabine caused a significant increase in the plasma levels of urea, creatinine and bilirubin (Table 3) of 28.98%, 23.60% and 34.83%, respectively, as compared with the control group. However, treatment with caffeic acid significantly ameliorated the capecitabine-induced increase in plasma activities of ALT, AST, ALP, and plasma levels of urea, creatinine and bilirubin in rats.

### 3.2. Effect of Caffeic Acid on Capecitabine-Induced Changes in the Activities of Enzymatic Antioxidants in the Liver of Rats

**Figure 2:**
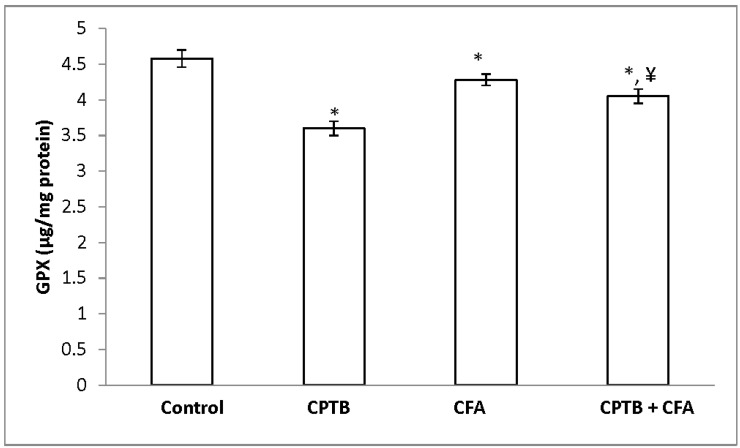
Protective effect of caffeic acid on the reduction of glutathione peroxidase (GPx) levels in rats due to capecitabine-induced toxicity. Data are expressed as means ± SD for six rats in each group; * significantly different from the control (*p* < 0.05); ^¥^ significantly different from the capecitabine group (*p* < 0.05).

**Figure 3:**
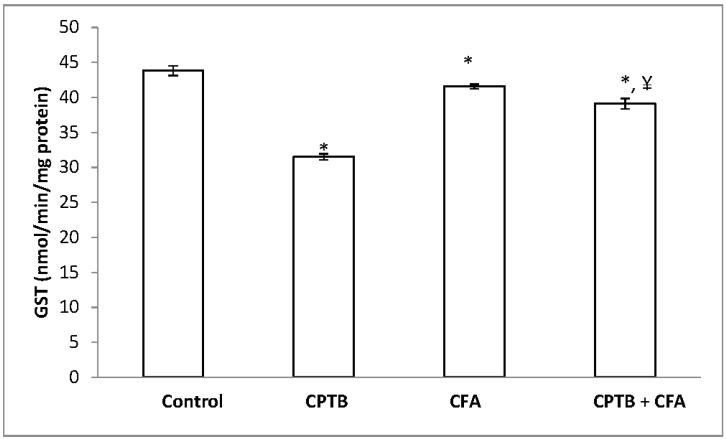
Influence of caffeic acid on capecitabine-induced changes in hepatic GST activity in rats. Data are expressed as means ± SD for six rats in each group; * significantly different from the control (*p* < 0.05); ^¥^ significantly different from the capecitabine group (*p* < 0.05).

Table 4 represents the protective effect of caffeic acid on capecitabine-induced reduction in the activities of SOD and CAT in the liver of rats. Hepatic SOD and CAT activities were significantly reduced (by 46.46% and 44.67%, respectively) in the capecitabine-treated group, when compared with values of the control group. Hepatic GPx and GST activities were also significantly reduced (by 28.16% and 21.45%, respectively) in the animal group treated with capecitabine alone when compared with the control group (Figure 2 and Figure 3). However, treatment with caffeic acid significantly ameliorated the reduced activities of hepatic SOD, CAT, GST and GPx.

### 3.3. Effect of Caffeic Acid on Capecitabine-Induced Changes in the Levels of Non-Enzymatic Antioxidant in the Liver of Rats

**Figure 4:**
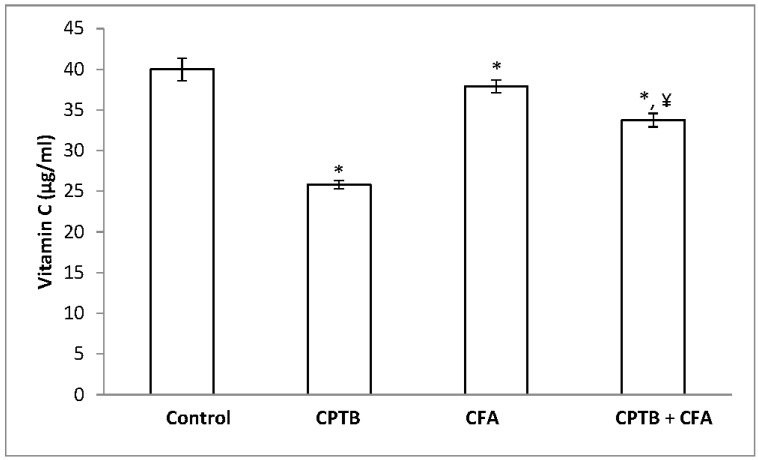
Protective effect of caffeic acid on the reduction of hepatic ascorbic acid (vitamin C) levels in rats due to capecitabine-induced toxicity. Data are expressed as means ± SD for six rats in each group; * significantly different from the control (*p* < 0.05); ^¥^ significantly different from the capecitabine group (*p* < 0.05).

**Figure 5:**
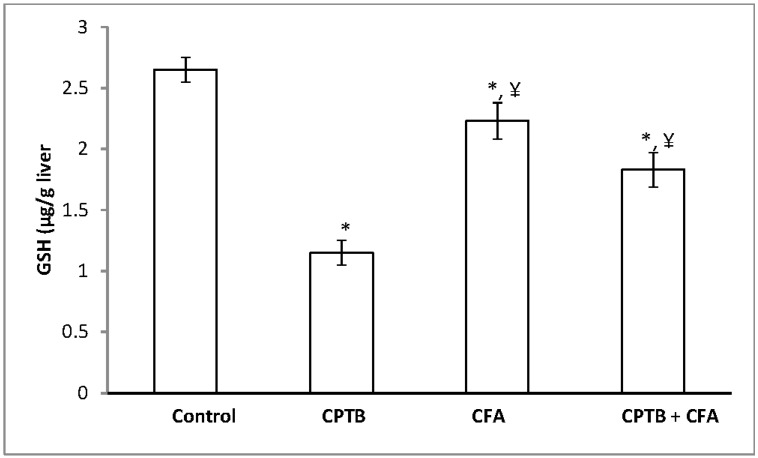
Protective effect of caffeic acid on the reduction of hepatic reduced glutathione (GSH) in rats due to capecitabine-induced toxicity. Data are expressed as means ± SD for six rats in each group; * significantly different from the control group (*p* < 0.05); ^¥^ significantly different from the capecitabine group (*p* < 0.05).

A significant reduction in the levels of hepatic AA and GSH (by 35.42% and 56.60%, respectively) was observed following the oral administration of capecitabine to rats when compared with the control group (Figure 4 and Figure 5). However, the level of these non-enzymatic antioxidants was enhanced following treatment with caffeic acid.

### 3.4. Influence of Caffeic Acid on Capecitabine-Induced Hepatic Lipid Peroxidation in Rats

**Figure 6:**
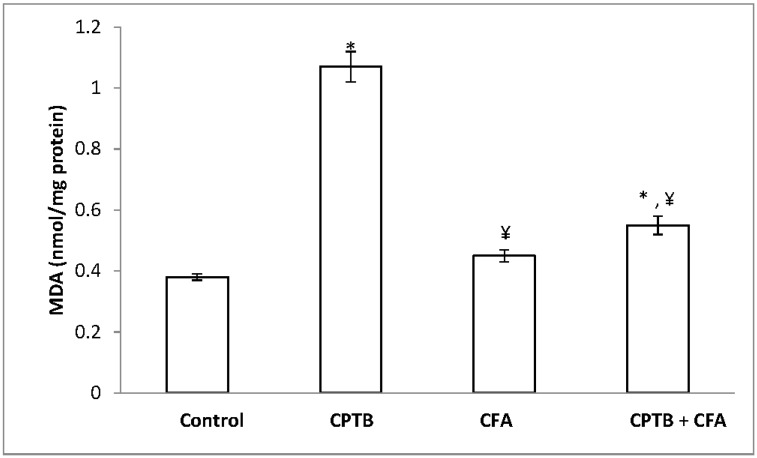
Protective effect of caffeic acid on the increase in lipid peroxidation (MDA) levels in rats due to capecitabine-induced toxicity. Data are expressed as means ± SD for six rats in each group; * significantly different from the control group (*p* < 0.05); ^¥^ significantly different from the capecitabine group (*p* < 0.05).

Figure 6 presents the protective effect of caffeic acid on capecitabine-induced hepatic lipid peroxidation. The level of malondialdehyde was significantly increased (by 64.22%) in the capecitabine-treated group when compared with the control (*p* < 0.05). However, treatment with caffeic acid significantly attenuated this increase in hepatic MDA relative to capecitabine group.

## 4. Discussion

It is well known that most drugs, especially chemotherapeutic agents, mediate their toxic effect through the generation of reactive oxygen species [2]. The recent report on the administration of capecitabine revealed that it could damage liver cells [10]. Reviews of several studies showed that caffeic acid and its modified ester forms exhibited excellent antioxidant capacities in diverse mechanisms [20,22,23]. This is the major reason why the present study was focused on the protective effect of caffeic acid against capecitabine-induced oxidative damage to liver organ in rats.

Reports have shown that the liver, which is a metabolically active organ, is an important target organ for most xenobiotics and environmental toxicants [35]. Specific catalytic proteins such as ALT, AST and ALP in blood serum are considered to be important biomarkers for liver dysfunction because their leakage from hepatocytes into blood are relevant biochemical indices for liver organ damage [36,37]. AST is present in high quantities in liver tissues relative to other tissues, such as kidney tissues. ALT is a principal cytoplasmic enzyme that is specific to the liver organ [38] and their leakage into circulation leads to increased plasma levels of ALT under pathological conditions such as hepatocellular injuries or inflammation of biliary tract cells [39,40]. Plasma levels of bilirubin and the ALP activities have been shown to increase in conditions associated with hepatobiliary injury and overproduction or leakage of ALP [41]. The results of this study suggest marked liver damage elicited by significant increases in levels of AST, ALT, ALP and bilirubin observed in an animal group treated with capecitabine. This result is in agreement with findings of other research on capecitabine administration in both rats and humans [10,42]. Further to this result, administration of caffeic acid offered ameliorative effects on capecitabine-induced hepatic injuries in rats. Our findings agreed with previous reports on the hepatoprotective activities of caffeic acid in rodents [43,44]. This reduction of hepatic injury confirms that caffeic acid has the capacity to maintain the normal architectural structure and integrity of hepatocytes, thereby preventing leakages of the serum marker enzymes. Kidney functions include excreting waste products of metabolism, such as ammonia, urea and creatinine. Reliable evidence of acute kidney dysfunction has been associated with elevated plasma urea and creatinine levels [45]. The observed significantly elevated level of urea and creatinine in capecitabine-treated rats relative to the control group could be related with renal overload. This is because an increase in plasma levels of urea and creatinine is an indicator of acute renal failure, renal overload or an increase in protein catabolism [46].

Research on hepatoprotection has shown that phenolic compounds possess potent antioxidant activity against free radicals in living systems, offering protection against oxidative stress and liver damage in rats [47]. Hydroxycinnamic compounds, specifically caffeic acid, have displayed promising potential as a secondary metabolite in the detoxification of free radicals and protection against the generation of reactive oxygen species [15,48]. The decreased levels of antioxidants in living systems have been considered to be an important risk factor for the development of toxic liver injury [49]. In nature, the body uses different mechanisms, which include the activities of chain reaction terminators and scavengers of free radicals like vitamin C, vitamin E, CAT, SOD and GSH, to protect itself from toxic effects of reactive oxygen species (ROS) [50].

CAT and SOD are intracellular enzymes which form the primary defence system against oxidative stress [51]. SOD catalyses the dismutation reaction that converts the superoxide ion into hydrogen peroxide, which is rapidly removed by the catalase enzyme that turns it into water and oxygen [52,53]. The levels of these enzymes are high in liver cells and erythrocytes [51]. The result of this study showed a significant reduction in the activities of CAT and SOD in the liver homogenate of the animal group treated with capecitabine alone, relative to the control animal group. The fact that catalase removes H_2_O_2_ that is generated in the liver cells by SOD suggests that accumulation of H_2_O_2_ might be responsible for the inactivation of SOD, which is confirmed by its concomitant low levels observed in this study. Moreover, the significant decrease in the levels of CAT and SOD may influence the susceptibility of the liver to oxidative stress induced by H_2_O_2_, hydroxyl radicals and the superoxide ion [54,55]. However, in this study, administration of caffeic acid offered significant protection by raising the levels of both SOD and CAT to near the normal levels of the control animals.

The assessment of GSH and ascorbic acid in living systems provides a prediction of cellular redox status. This is because GSH and AA have the capability to scavenge free radicals and act as the first line of defence against cellular oxidation [56,57]. GST is an enzymatic antioxidant that detoxifies most highly reactive electrophiles, carcinogens and products of oxidative stress in conjugation with GSH [58]. The importance of the GSH molecule has been linked to its capacity to regenerate active forms of both vitamins C and E, and with the detoxification of endogenous and exogenous toxic substances in the body [59,60]. In the present study, a significant reduction in the levels of GSH and vitamin C, and in the activities of GST and GPx in the animal group treated with capecitabine was observed relative to the control group. The reduction in the level of GST in this study correlates with a depletion of GSH levels in the liver, which confirms that capecitabine is toxic. Additionally, the decrease in GST with the concomitant depletion of GSH has been associated with severe oxidative stress [61]. However, administration of caffeic acid significantly improved the redox balance in the liver of the rats by the significant increase observed in the levels of GSH and vitamin C, and in the activities of GST and GPx. This could be due to the antioxidant potential of caffeic acid. This observation is in agreement with results obtained from various in vivo studies on caffeic acid and its derivatives [62]. Furthermore, there was a significant increase in the activities of GPx observed in the animals treated with caffeic acid relative to the capecitabine group. GPx is an enzyme that reduces the reactive peroxides to alcohols and water with the resultant oxidation of GSH to GSSG. This study suggests that capecitabine can enhance the generation of reactive peroxide as part of its mechanisms of toxicity due to a reduction in GPx activity, and caffeic acid has the capacity to facilitate the removal of peroxide by raising the levels of GPx in the liver of rats.

In the present work, the administration of capecitabine induced oxidative stress in rat livers as evidenced by perturbation in both enzymatic and non-enzymatic antioxidative markers. This shows that the pathogenesis of capecitabine-induced liver toxicity is mediated by reactive oxygen species. Lipid peroxidation, which is a molecular mechanism of the oxidation of cellular lipids, is an accepted indicator of oxidative stress resulting from overwhelming ROS and depleted antioxidant levels [63]. The presence of polyunsaturated fatty acid tails on phospholipid components in biological membranes has been shown to be responsible for the fluid property of those membranes [64]. Excess free radicals attack unsaturated fatty acid and initiate lipid peroxidation, which leads to a complex series of compounds like MDA, reactive carbonyl compounds and lipid peroxides [53,65]. More commonly, increased MDA levels are considered to be a reliable indicator of enhanced lipid peroxidation that depicts tissue damages [66]. The apparent increase in malondialdehyde levels observed in animals treated with capecitabine alone, relative to the control group, indicates that capecitabine could induce oxidative stress through enhanced lipid peroxidation. However, the result of this work further showed that the increased level of MDA in the rats was significantly attenuated by caffeic acid.

## 5. Conclusions

The present work demonstrated that caffeic acid has the capacity, through the enhancement of endogenous enzymatic and non-enzymatic antioxidants, to protect against hepatic and renal dysfunction, and oxidative stress induced by capecitabine chemotherapy. It is therefore suggested that caffeic acid, when used as an auxiliary therapy in cancer treatment, may mitigate the adverse effects of oxidative stress posed by chemotherapies such as capecitabine. However, further studies may be required to ascertain any possible influence of caffeic acid on the chemotherapeutic activity of capecitabine.

## Figures and Tables

**Table 1:** Experimental design.

Treatment Groups	Treatment Duration (1–14 Days)
A. Control	Distilled water
B. Capecitabine (CPTB)	30 mg/kgbw CPTB
C. Caffeic acid (CFA)	100 mg/kgbw CFA
D. CPTB + CFA Co-treated	100 mg/kg bw CFA + 30 mg/kgbw CPTB

**Table 2:** Protective effects of caffeic acid on capecitabine-induced changes in the plasma activities of alanine aminotransferase (ALT), aspartate aminotransferase (AST), and alkaline phosphatase (ALP) in rats.

Treatment	AST (U/L)	ALT (U/L)	ALP (U/L)
Control	46.5 ± 1.90	3.15 ± 0.11	436 ± 2.34
CPTB	74.8 ± 1.20 (37.86%) *	6.86 ± 0.15 (54.13%) *	655 ± 4.82 (33.40%) *
CFA	50.7 ± 1.21 *	3.63 ± 0.12 *	460 ± 7.36 *
CPTB + CFA	59.5 ± 3.10 *^,¥^	4.48 ± 0.16 *^,¥^	540 ± 6.64 *^,¥^

Data represent the means ± standard deviation (SD) for six rats in each group; * significantly different from the control group; ^¥^ significantly different from capecitabine group (*p* < 0.05). Values in parenthesis represent a percentage (%) increase.

**Table 3:** Protective effects of caffeic acid on capecitabine-induced changes in the plasma levels of urea, creatinine and bilirubin in rats.

Treatment	Urea (mg/dL)	Creatinine (mg/dL)	Bilirubin (mg/dL)
CONTROL	74.33 ± 2.34	0.98 ± 0.02	3.15 ± 0.1
CPTB	104.7 ± 1.03 (28.98%) *	1.28±0.01 (23.60%) *	4.83 ± 0.1 (34.83%) *
CFA	78.8 ± 1.47 *	1.10 ± 0.02 *	3.48 ± 0.2 *
CPTB + CFA	90.0 ± 2.28 *^,a^	1.15 ± 0.01 *^,a^	3.75 ± 0.1 *^,a^

Data represent the means ± standard deviation (SD) for six rats in each group; * significantly different from the control group. Values in parenthesis represent a percentage (%) increase

**Table 4:** Protective effect of caffeic acid on capecitabine-induced changes in activities of hepatic superoxide dismutase (SOD) and catalase in rats.

Treatment	SOD (Units)	Catalase (µmole H_2_O_2_ Consumed/min/mg protein)
Control	26.3 ± 1.37	1.48 ± 0.02
CPTB	14.1 ± 0.65 (46.46%) *	0.79 ± 0.04 (44.67%) *
CFA	23.6 ± 0.89 *	1.23 ± 0.03 *
CPTB + CFA	19.7 ± 0.82 *^,¥^	1.10 ± 0.06 *^,¥^

Data are expressed as means ± SD for six rats in each group; * significantly different from the control group (*p* < 0.05); ^¥^ significantly different from the capecitabine group (*p* < 0.05). Values in parenthesis represent percentage decrease.
